# Isolation of *Mycobacterium massiliense* from a corneal biopsy in India

**DOI:** 10.1099/jmmcr.0.003350

**Published:** 2014-12-01

**Authors:** Lily Therese Kulandai, Dhanurekha Lakshmipathy, Gayathri Ramasubban, Madhavan Hajib Narahari Rao

**Affiliations:** L&T Microbiology Research Centre, Vision Research Foundation, New No. 41, Old No. 18, College Road, Chennai – 600006, India

**Keywords:** corneal biopsy, *Mycobacterium massiliense*, RGM

## Abstract

**Introduction:**

Rapidly growing mycobacteria (RGM) are ubiquitous and are usually considered as saprophytes, and have been recovered from the environment, particularly in dust, watery soil and water distribution systems. However, *Mycobacterium massiliense* is a rare causative agent of ocular infection.

**Case presentation:**

We report a case of *M. massiliense* in a 44-year-old female with signs and symptoms of a corneal ulcer. We carried out PCR-based DNA sequencing targeting the *hsp 65* gene for the identification of *M. massiliense*. To confirm the identification, we also performed PCR-based RFLP targeting the *hsp65* gene and PCR-based DNA sequencing targeting the internal transcribed spacer region, which showed 97 % nucleotide identity with *M. massiliense*.

**Conclusion:**

To the best of our knowledge, this is the first study in India to report the detection of *M. massiliense* from a corneal biopsy.

## Introduction

Rapidly growing mycobacteria (RGM) are ubiquitous and are usually considered saprophytes, and have been recovered from the environment, particularly in dust, watery soil and water distribution systems. RGM are currently gaining attention because of their emerging importance in both sporadic infection and outbreak settings (Adekambi *et al*., 2004). The most common RGM causing human diseases are *Mycobacterium abscessus*, *Mycobacterium chelonae* and *Mycobacterium fortuitum*. RGM are rare causative agents of ocular infection and are associated with ocular trauma, contact lens use and corneal procedures especially laser *in situ* keratomileusis, extraocular surgery including tear duct probing, scleral buckling and dacryocystorhinostomy intraocular surgery including cataract surgery, penetrating keratoplasty and intravitreal triamcinolone injection.

*Mycobacterium massiliense* is a rapidly growing *Mycobacteria* species sharing an identical 16S rRNA gene sequence with *Mycobacterium abscessus*. The species *M. massiliense* was proposed in 2004 and the name was validated in 2006 (Simmon *et al*., 2007). They are strictly aerobic, non-motile, non-spore-forming, acid-fast, Gram-positive rods. In contrast to *Mycobacterium tuberculosis*, there is no systematic reporting of non-tuberculous mycobacterial infections; thus, precise incidence data are lacking. To the best of our knowledge, this is the first report on the isolation of *M. massiliense* from a corneal biopsy in India.

## Case report

A corneal biopsy specimen from a 44-year-old female with signs and symptoms of corneal ulcer was received in the microbiology laboratory for mycobacteriology investigations. Acid-fast staining by the Ziehl–Neelsen method showed the presence of acid-fast bacilli. Culture for acid-fast bacilli was done using a BACTEC Micro MGIT culture system (Becton Dickinson, Maryland, USA), which showed a positive signal after 5 days, and the bacteria were subsequently subcultured on to Löwenstein–Jensen (LJ) medium in triplicate and on blood agar and MacConkey agar. The culture grew at 25 and 37 °C but not at 42 °C on LJ medium, blood agar and MacConkey agar but not on LJ medium containing 4.5 % NaCl. The conventional biochemical tests for iron uptake, aryl sulphatase (3 days) and nitrate reductase were positive, whilst aryl sulphatase (7 days) was negative.

After the culture became positive, nested PCRs targeting the *MPB64* (Therese *et al*., 2005) gene and the IS*6110* (Therese *et al*., 2005) region for detection of the *M. tuberculosis* genome were performed on the isolate and were negative, indicating that the isolate was a non-tuberculous mycobacterium.

To confirm the identification, PCR-based RFLP (Wang *et al*., 2004) using the enzymes *Bst*EII and *Hae*III targeting the *hsp65* gene and PCR-based DNA sequencing targeting the *hsp65* and internal transcribed spacer (ITS) (Telenti *et al*., 1993) region were performed.

**Fig. 1.**
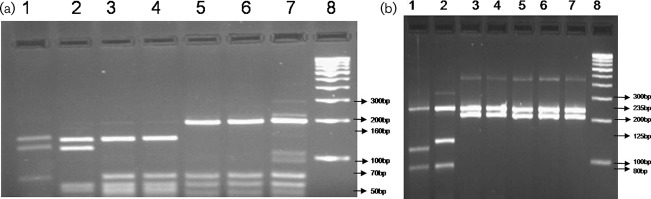
PCR RFLP patterns of *M. massiliense* digestion with *Hae*III and *Bst*EII. (a) *Hae*III digestion. Lanes: 1, *M. tuberculosis* H37RV ATCC (160, 140, 70 bp); 2, *M. fortuitum* ATCC 1529 (155, 135 bp); 3, *M. chelonae* TMC 1542 (160, 60 bp); 4, *M. abscessus* laboratory isolate (160, 60 bp); 5, *M. massiliense* isolate from corneal biopsy (this study; 200, 70, 60, 50 bp); 6 and 7, *M. massiliense* isolate from non-ocular specimens (not included in this paper); 8, 100 bp molecular weight ladder. (b) Bst*EII* digestion. Lane: 1, *M. tuberculosis* H37RV ATCC (245, 120, 80 bp); 2, *M. fortuitum* ATCC 1529 (245, 125, 80 bp); 3, *M. chelonae* TMC 1542 (245, 220 bp); 4, *M. abscessus* lab isolate (245, 220 bp); 5, *M. massiliense* isolate from corneal biopsy (235, 220 bp); 6 and 7, *M. massiliense* isolate from non-ocular specimens (not included in this paper); 8, 100 bp molecular weight ladder.

PCR-based RFLP using *Bst*EII and *Hae*III yielded fragments of 235 and 220 bp, and of 200, 70, 60 and 50 bp, respectively (Fig. 1). PCR-based DNA sequencing targeting *hsp65* and the ITS region show 97 % nucleotide identity with *M. massiliense* reference strains deposited in GenBank and our *M. massiliense* sequence was deposited in GenBank. Antibiotic susceptibility testing was performed using the Kirby–Bauer method, and the isolate was found to be sensitive to cefoperazone, doxycycline, cefotaxime and clarithromycin and resistant to cefuroxime, ceftriaxone, ceftazidime and cefazolin.

## Discussion

*M. massiliense* has recently been identified as a new species of non-tuberculous RGM causing human infections. Recently, reports on RGM infections in various clinical situations have markedly increased, and in these reports, *M. abscessus* infection is the most frequently encountered. Moreover, nearly 95 % of soft-tissue infections caused by RGM are *M. chelonae*–*M. abscessus* complex infections (Simmon *et al*., 2007). *M. chelonae*–*M. abscessus* group taxonomy has undergone several updates due to the discrimination of new species by sequencing multiple housekeeping genes and, to a lesser extent, by the evaluation of phenotypic characteristics. *M. massiliense* is a newly proposed species that is closely related to members of the *M. abscessus*–*M. chelonae* group, and consequently should be considered part of the same group.

*M. massiliense* was first reported to cause human infection by Adekambi *et al*. (2004) following its isolation from the lower respiratory tract of a 50-year-old woman with haemoptoic pneumonia. Kim *et al*. (2007) reported an outbreak of *M. massiliense* infection associated with intramuscular injections administered at a local clinic in Korea, and carried out a comparative sequence analysis of the 16S rRNA, *rpoB* and *hsp65* genes by PCR-based DNA sequencing and also constructed phylogenetic trees obtained from the *rpoB* and *hsp65* sequences. Simmon *et al*. (2007) described the isolation and identification of *M. massiliense* in the USA associated with invasive infections, and sequenced portions of the *rpoB*, *sodA* and *hsp65* genes to gain a better understanding of the frequency of detection of *M. massiliense* or *Mycobacterium bolletii* among clinical isolates identified as being *M. chelonae/M. abscessus* by 16S rRNA and ITS assays. Recently, Cristina *et al.* (2008) suggested a newer classification of *M. chelonae*–*M. abcessus* group that included *M. chelonae*, *Mycobacterium immunogenum* and *M. abscessus* with *M. abscessus* subsp. *abscessus* and *M. abscessus* subsp. *massiliense* using both phenotypic identification (biochemical tests, high-performance liquid chromatography and drug susceptibility testing) and genotypic identification (DNA sequencing and phylogenetic analysis using the *hsp65* and *rpoB* genes, PCR/restriction enzyme analysis for *hsp6*, and RFLP analysis of the 16S rRNA gene) (Cristina *et al*., 2008). In our study, we carried out PCR-based DNA sequencing targeting the *hsp65* gene for the identification of *M. massiliense*. To confirm the identification, we also performed PCR-based RFLP targeting *hsp65* and PCR-based DNA sequencing targeting the ITS region showing 97 % nucleotide identity with *M. massiliense*. In conclusion, we believe that this is the first report on the isolation of *M. massiliense* from a corneal biopsy in India, and *M. massiliense* should be considered an emerging pathogen, but further studies should be made to understand the pathogenic mechanism of this organism.

## Abbreviations

ITS: internal transcribed spacer
RGM: rapidly growing mycobacteria
